# SORGOdb: Superoxide Reductase Gene Ontology curated DataBase

**DOI:** 10.1186/1471-2180-11-105

**Published:** 2011-05-16

**Authors:** Céline Lucchetti-Miganeh, David Goudenège, David Thybert, Gilles Salbert, Frédérique Barloy-Hubler

**Affiliations:** 1 CNRS UMR 6026, ICM, Equipe Sp@rte, Université de Rennes 1, Campus de Beaulieu, 35042 Rennes, France; 2 EMBL-EBI Wellcome Trust Genome Campus; Hinxton, Cambridgeshire, CB10 1SD, UK

## Abstract

**Background:**

Superoxide reductases (SOR) catalyse the reduction of superoxide anions to hydrogen peroxide and are involved in the oxidative stress defences of anaerobic and facultative anaerobic organisms. Genes encoding SOR were discovered recently and suffer from annotation problems. These genes, named *sor*, are short and the transfer of annotations from previously characterized neelaredoxin, desulfoferrodoxin, superoxide reductase and rubredoxin oxidase has been heterogeneous. Consequently, many *sor *remain anonymous or mis-annotated.

**Description:**

SORGOdb is an exhaustive database of SOR that proposes a new classification based on domain architecture. SORGOdb supplies a simple user-friendly web-based database for retrieving and exploring relevant information about the proposed SOR families. The database can be queried using an organism name, a locus tag or phylogenetic criteria, and also offers sequence similarity searches using BlastP. Genes encoding SOR have been re-annotated in all available genome sequences (prokaryotic and eukaryotic (complete and in draft) genomes, updated in May 2010).

**Conclusions:**

SORGOdb contains 325 non-redundant and curated SOR, from 274 organisms. It proposes a new classification of SOR into seven different classes and allows biologists to explore and analyze *sor *in order to establish correlations between the class of SOR and organism phenotypes. SORGOdb is freely available at http://sorgo.genouest.org/index.php.

## Background

Two and a half billion years ago, the intense photosynthetic activity of *cyanobacteria *caused the largest environmental change in Earth's history: the oxygenation of the atmosphere and the oceans, which were hitherto largely anoxic [1,2]. This profound transformation of the biosphere exerted an evolutionary selection pressure on organisms and led to the development of new pathways, including the highly exergonic respiratory chain based on O_2 _as the terminal electron acceptor. Currently, most living organisms, except anaerobic microbes, require oxygen. O_2 _is used as a substrate by many enzymes involved metabolizing amines, purines and amino acids. Oxygen is a relatively inert molecule due to its spin triplet ground state. However, it can be activated by photons or by one electron oxidation or reduction processes to generate reactive oxygen species (called reactive oxygen species or ROS), particularly hydroxyl radicals (•OH), hydrogen peroxide (H_2_O_2_) and superoxide anion radicals (O_2_-).

The superoxide anion is generated fortuitously by flavoenzymes such as NADH dehydrogenase II, succinate dehydrogenase, fumarate reductase, and sulphite reductase [3,4]. The superoxide anion is one of the deleterious reactive oxygen species: it can damage DNA, proteins and lipids indirectly by releasing iron from damaged dehydratase clusters [4,5]. In anaerobes, most of the essential "central metabolic" redox enzymes (for example aconitase, fumarase, dihydroxyacid dehydratase, and pyruvate:ferredoxin oxidoreductase) contain iron sulphur [Fe-S] clusters that are rapidly inactivated when exposed to oxygen [5-8].

To survive and protect themselves from the toxicity of superoxide anion, many species, and especially anaerobes, have developed defence mechanisms [5].

Superoxide dismutase (SOD) was first isolated by Mann and Keilis (1938) and its catalytic function, which consists to dismutate O_2_- into molecular oxygen and hydrogen peroxide, was discovered in 1969 by McCord and Fridovich [9]. Mammals have two forms of SOD isozymes: the manganese SOD (Mn-SOD), present in the mitochondria, and the copper/zinc SOD (Cu/Zn-SO), present in the cytoplasm [10,11]. In plants, SOD have been classified into three distinct types on the basis of their metal cofactor: Cu/Zn-SOD (in the cytosol and chloroplasts), Mn-SOD (in mitochondria), and Fe-SOD (often in chloroplasts) [12-14]. There are three known SOD in *E. coli*: MnSOD, FeSOD and CuZnSOD. The two first are located in the cytoplasm and the last in the periplasmic space [15]. A distinct additional fourth class of SOD containing nickel (NiSOD) was recently discovered in Streptomyces [16,17] and cyanobacteria [18]. SOD-driven dismutation was the only biological mechanism identified for scavenging superoxide anion radicals until the early 1990's. McCord *et al*. [19] established a correlation between oxygen tolerance and SOD production and suggested that SOD was the single most important enzyme for enabling organisms to survive in the presence of molecular oxygen. They proposed that the hypersensitivity of obligate anaerobes to oxygen was a consequence of SOD deficiency. However, most anaerobic organisms, which indeed lack SOD, show various degrees of tolerance to oxygen when they are occasionally exposed to this molecule in their environments.

Two novel iron-sulphur-containing proteins that detoxify superoxide molecules were then discovered in sulphate-reducing and hyperthermophilic anaerobes: desulfoferrodoxin (Dfx) in *Desulfovibrio desulfuricans, Desulfovibrio vulgaris Hildenbourgh *[20] and *Desulfoarculus baarsii *[21], neelaredoxin (Nlr) in *Desulfovibrio gigas *[22] and superoxide reductase (SOR) in *Pyrococcus furiosus *[23]. This revealed the existence of alternative mechanisms for ROS detoxification in anaerobes. The function of these proteins was first studied in 1996 by Dfx complementation of superoxide detoxication activity in *E. coli *SOD mutants [24]. Later, Nlr from *Treponema pallidum *[25] and *D. gigas *[26] were also shown to complement such SOD mutants. Liochev and Fridovich [27] suggested that Dfx catalyzes the reduction of superoxide rather than its dismutation, and that it uses cellular reductants such as NAD(P)H. Subsequently, the Dfx enzyme was confirmed as an oxidoreductase [23-25,27]. Finally, the superoxide reductase activity of those proteins were established by two groups [21,23].

Dfx and Nlr proteins have different numbers of iron sites: both contain a similar C-terminal single iron-containing site (centre II) but also has Dfx a second N-terminal site (centre I) [22,28]. Centre II is the active site of SOR and consists of a pentacoordinated Fe^2+ ^centre with four equatorial histidines and one axial cysteine in a square pyramidal geometry (Fe(His)_4_(Cys) [29-31]). The binding site for the substrate O_2_- is the free sixth axial site of the reduced enzyme centre [30]. The additional N-terminal domain of the 2Fe-SOR contains a rubredoxin-like centre, with Fe^3+ ^ligated by four cysteines in a distorted tetrahedral geometry (centre I, Fe(Cys)_4_, [32]). A first classification of these enzymes was proposed according to the number of metal centres: neelaredoxin or 1Fe-SOR and desulfoferrodoxin or 2Fe-SOR [33,34]. An additional class was proposed after the isolation of a *Treponema pallidum *SOR that contains an extended non-iron N-terminal domain of unknown function [25,35]. In all these three classes, only the reduced form of the iron-containing active centre II is able to react with the superoxide anion O_2_•^-^.

SOD are found in nearly every living organism except in some strictly anaerobic species [36,37]. Tally *et al *suggested that the diversity in the oxygen tolerance of anaerobes is generally related to their level of SOD [38]. SOR were first thought to be restricted to anaerobic prokaryotes but were subsequently discovered in some micro-aerophilic and micro-aerotolerant Bacteria and Archaea [39,40]. More recently, a SOR encoding gene was also discovered in an eukaryote, *Giardia intestinalis*, a microaerophilic protozoan (cited by [41]). Although SOD and SOR both detoxify superoxide, there is a fundamental difference in their properties: SOD generate one-half mole of oxygen and one-half mole of hydrogen peroxide per superoxide molecule whereas SOR produce only one mole of hydrogen peroxide. The physiological conditions, that determine SOR or SOD preference in organisms, have not be completely determined, although the presence of SOR rather than SOD may be associated with the amount of redox proteins produced by organisms [25].

Most genomes, even those of anaerobic species, contain both SOD and SOR although some species have only one of the two enzymes. The increasing number of sequenced genomes makes allows comparative genomic analyses, to elucidate the evolutionary or functional processes of SOR. Unfortunately, there are several problems with the annotation of superoxide reductase genes, partly a consequence of heterogeneous transfer of annotations from previously characterized neelaredoxin, desulfoferrodoxin, superoxide reductase or rubredoxin oxidase. Moreover, due to the absence of updating or correction of databases, many *sor *genes remained anonymous because of the transfer of annotations from SOR genes initially annotated as "hypothetical", "function unknown" or "putative activity". Also, SOR are small proteins, ca. 200 amino acids on average, and mis-annotations are frequent for proteins of this length [42].

For all these reasons, we developed SORGOdb, the first resource specifically dedicated to superoxide reductase genes in entirely sequenced and in-draft genomes. SOR sequences were curated manually, analysed and stored using a new ontology in a publically available resource (http://sorgo.genouest.org/). SOR genes were detected in the three kingdoms of life, and only on chromosomal replicons. Although no N-terminal signal sequences were previously described for bacteria SOR [43], we predicted seven SOR to be potentially TAT-secreted (Twin-arginine translocation) in some bacteria, including for example in *Desulfovibrio salexigens DSM 2638, Desulfuromonas acetoxidans DSM 684 *and *Geobacter uraniireducens Rf4*. Our analysis confirms the observations by Pinto *et al *in 2010 that (1) the repartition of SOR classes does not correlate with organism phylogeny and that (2) *sor *genes occur in very diverse genetic environments. Indeed, although some *sor *are clustered with genes encoding electron donors (such as rubredoxin in *D. vulgaris*) or inter-related oxidative responsive genes, most are close to functionally unrelated genes. This is consistent with *sor *genes being acquired, or lost, through lateral gene transfer [41].

## Construction and content

### Collection of SOR

For collection of SOR, we have extensively searched the Pubmed database and identified all relevant literature concerning any protein with "superoxide reductase" activity; this search resulted in a small dataset (13 SOR published in 12 organisms, see Table 1). We therefore enriched the database using manually curated sequences described as desulfoferrodoxin (160 proteins), superoxide reductase (50 proteins) or neelaredoxin (9 proteins) in EntrezGene and/or GenBank entries. As the "centre II" is the active site for the SOR activity, we also included all proteins with a domain of this type as described in InterPro (IPR002742, IPR004793, IPR004462, IPR012002), Pfam (PF01880, PF06397), Supfam (SSF49367), TIGRfam (TIGR00332, TIGR00320, TIGR00319), NCBI conserved domains (cd03172, cd03171, cd00524, cl00018, cl00014, cd00974) and PRODOM (PD006618, PD330262, PDA2O7Z7, PDA36750, PD985590, PDA36751, PDA63215, PDA7Y161, PDA7Y162, PD511041, PD171746, PD985589, PDA7Y163). All sequences collected were cleaned up to remove redundancy and unrelated proteins. This non-redundant and curated dataset was used to investigate the 1237 complete and 1345 in-draft genomes available in the NCBI database (May, 2010) through a series of successive BlastP [44] and tBlanstN [45] searches. Orthology (KO K05919 and COG2033) and synteny (IMG neighbourhood interface) were also exploited. To be as comprehensive as possible in the data collection, we performed multiple alignments using both ClustalW [46,47] and Muscle [48] algorithms. These alignments showed highly conserved residues in the sequences of active centre I (CX_2_CX_15_CC) and centre II (HX_5_H-CX_2_H ). These conversations were translated into "regular expressions" that were used to perform for final screening of databases. All these search processes allowed us to retrieve 106 supplementary proteins including 82 proteins described as "hypothetical protein".

**Table 1:** SOR proteins with entrie(s) in Pubmed and/or PDB structure

Organism	Locus Tag	PDB	PMID
*Desulfovibrio desulfuricans ssp. desulfuricans. ATCC 27774*	Ddes_2010	1DFX	[20,56,76-78]
*Desulfovibrio Desulfuricans ssp. desulfuricans G20*	Dde_3193	2JI3, 2JI2,	[79]
*Desulfoarculus baarsii*	rbo	2JI1, 1VZI, 1VZG, 1VZH	[25,52,79-87]
*Pyrococcus horikoshii Ot3*	PH1083	2HVB	[30]
*Pyrococcus furiosus DSM 3638*	PF1281	1DQI, 1DO6, 1DQK	[29,30,88-91]
*Treponema pallidum ssp. pallidum str. Nichols*	TP0823	1Y07	[21,35,52,82,86,92-99]
*Treponema maritima*		2AMU	
*Archaeoglobus fulgidus DSM 4304*	AF0833, AF0344		[51,55,100-103]
*Desulfovibrio vulgaris 'Miyazaki F*	DvMF_2481		[104]
*Desulfovibrio vulgaris sp. vulgaris str. Hildenborough*	DVU3183		[20,54,97,105-108]
*Desulfovibrio gigas*	nlr		[22,26,109]
*Clostridium acetobutylicum ATCC 824*	CAC2450		[110,111]
*Nanoarchaeum equitans Kin4-M*	NEQ011		[112]

PDB: Protein Data Bank (http://www.pdb.org/pdb/home/home.do)

PMID: PubMed unique identifier (http://www.ncbi.nlm.nih.gov/pubmed)

At the end of this integrative research, we had a collection of 325 non-redundant and curated predicted SOR in 274 organisms, covering all the three kingdoms: Bacteria (270 genes), Archaea (52 genes) and Eukaryota (3 genes).

### New Classification and ontology

Consistent with the collecting procedure, all the 325 proteins present in SORGOdb contain at least the SOR active centre II domain. However, we found that this SOR module is, in some cases, associated with other domains, in a modular way. The discovery of new combinations of domains makes the previous classification into three classes inappropriate. Indeed, we suggest that the existence of multi-domain SOR indicates new function due to cooperation between domains. As previously proposed, the concept of orthology is more relevant at the level of domains than at the level of whole proteins except for proteins with identical domain architectures [49,50]. We therefore propose a new unambiguous SOR classification based on their domain architectures (sequential order of domains from the N- to the C-terminus [49]). Considering both domain compositions and arrangements, this classification contains seven functionally relevant classes which were precisely described on the website (http://sorgo.genouest.org/classif.php, additional file 1 and Table 2). Briefly, the 144 proteins that contain only the active site II (SOR) without other additional domains or cofactors have been classified as Class II-related SOR and correspond to the previous SOR class II [20,22,23,51]. Class III-related SOR correspond to the previous SOR class III proteins which have the active site II and enclose an additional N-terminal region of unknown function [25,35,52]. Class-IV related SOR correspond to very recently new class of methanoferrodoxin [53] which have the active site II and an additional iron sulfur domain. The TAT-SOR have the active site II and include an extra twin-arginine N-terminal signal peptide. The 152 proteins composed of a desulforedoxin (Dx) domain preceding the SOR unit (formerly Class I [20,21,54-56]) were clustered in a class named Dx-SOR. The 19 proteins that combined a N-terminal helix-turn-helix domain (HTH) before the Dx-SOR module were gathered in a separate class called HTH-Dx-SOR. Finally, 10 SOR proteins that correspond to exceptional domains fusion or that encompass a mutated ncDx domain (frameshift or mutation in the conserved CXXCX15CC metal binding residues) were classified in a disparate class labelled "Atypical-SOR". This class is quite heterogeneous but includes all proteins whose composite or mutated structure might suggest a function different of the three previous classes or, in the case of mutants, a non-functionality due to the loss of key binding sites.

**Table 2:** Classes of SOR in SORGOdb (Number of proteins per classes)

**SOR in SORGOdb**	**Dx-SOR**	**SOR**	**HTH-SOR**	**Atypical SOR**
325	152	144	19	10

### SORGOdb website construction

SORGOdb is a relational database built on MySQL and accessed from a PHP web-based interface (phpMyAdmin, Ratschiller, 2000) with additional JavaScript and JQuery functionalities (Jquery JavaScript library released in 2006 by John Resig). The database runs with the Apache web server version 2.2.3, hosted at the BioGenouest bioinformatics platform (http://www.genouest.org/). The sequences, features and annotations were introduced into the database using Python-based scripts.

### SORGOdb Web interface

**Figure 1:**
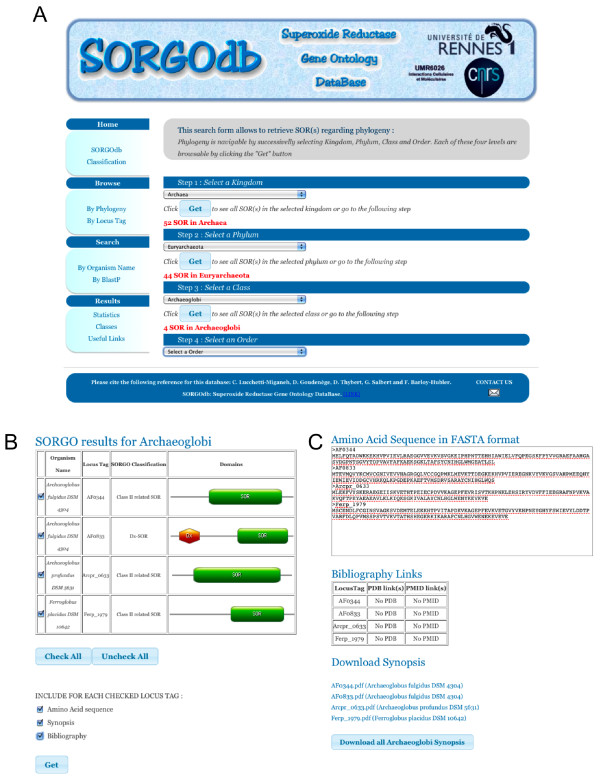
**A snapshot of the SORGOdb input interface**. **(A) **The "Browse By Phylogeny" module allows the selection of organisms with an SOR, using complete phylogeny criteria (kingdom, phylum, class and order). **(B) **The results panel provides intermediary selection options and displays SOR record information in a tabular way including organism name, locus tag name, SORGOdb classification and domain architecture. **(C) **Using checkboxes, amino acid sequences and bibliography links can be obtained and the synopsis can be downloading in .pdf format.

SORGOdb includes both documentation and search options. The web interface is composed of two panels (Figure 1).

The navigation menu (on the left) provides access to SORGOdb functions through three modules. (i) Browse: browse SOR proteins according to phylogeny criteria (kingdom, phylum, class and order) or locus tag name. (ii) Search: by organism name query and by sequence similarity through a BlastP form that allows users to enter primary sequences to find similar entries into the SORGOdb database and (iii) Pre-computed Results that include data statistics (organized in three tabs), classes (details about SORGOdb classes and ontology) and useful links (reference, tools and websites). Statistical results about SORGOdb classification were presented in the Classification tab (http://sorgo.genouest.org/classif-Stat.php).

The results panel (on the right) provides intermediary selection options and displays SOR record information in a tabular way including organism name, locus tag name, SORGOdb classification and domains architecture. When available, SORGOdb includes a CGView [57] representation of the distribution of SOR and all SOD genes (MnSOD, FeSOD CuZnSOD and NiSOD) [36] in the replicons and a gView [58] map to illustrate the genetic organisation and encoded functions surrounding each SOR (window of 11 genes max.).

### SORGOdb synopsis and download

**Figure 2:**
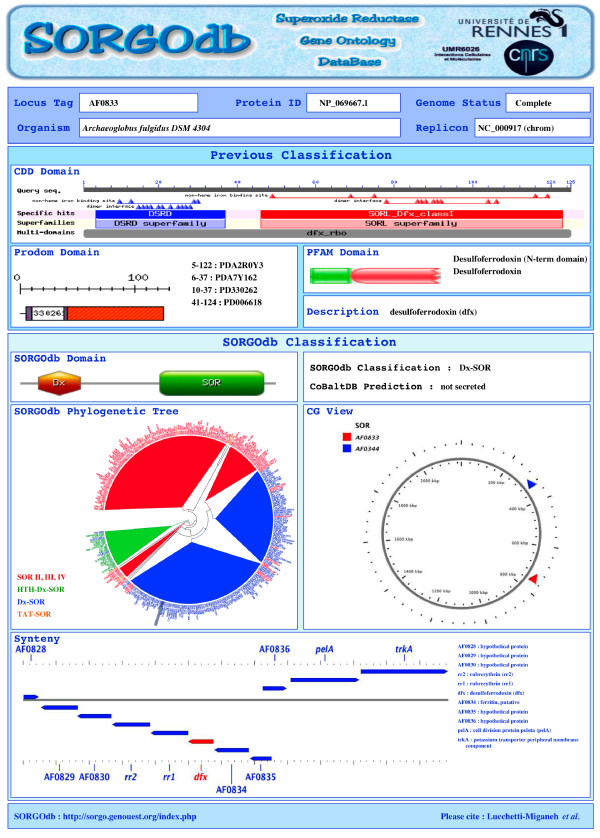
**SORGOdb Synopsis**. For any given protein, all results are summarized in a synopsis which presents results from disparate resources in an unified manner, and includes (i) the previous classification with the SOR description, the domain predictions (ii) the SORGOdb classification with domain representations, the SOR cellular localization prediction, the phylogenetic tree, the position of the *sor *gene and in some cases the *sod *gene on the replicon and the local synteny (iii) and bibliography and PDB links when available. This synopsis can be stored as a .pdf file.

Using checkboxes, amino acid sequences and bibliography links can be obtained and synopsis cart can be downloading in .pdf format (Figure 2). Synopsis were created and pre-computed for each SOR (using Python scripts and PHP library FPDF v1.6, http://www.fpdf.org/) in order to highlight key findings in an unified manner with all protein information (locus tag, ID, organism name, replicon and genome status), previous (PRODOM, PFAM and CDD) and new (SORGOdb) classification, position in the SORGOdb distance tree, SOR cellular localization prediction using CoBaltDB [59], genomic organisation for SOR and SOD *loci*, synteny viewer, PMID and PDB references. Images were generated using Python scripts from CGview (genomic map), MyDomains (SORGOdb domains representation), CDD, PFAM and PRODOM (database domains illustration), gView (synteny organisation) and from FigTree (for distance tree; http://tree.bio.ed.ac.uk/software/figtree).

## Utility and Dicussion

As an example, SORGOdb allows the study of the distribution of genes encoding superoxide reductase across a whole phylum. As a case study, we decided to consider the Archaea as these organisms are considered to be originate from a hyperthermophilic anaerobic common ancestor and were probably already prevalent when the Earth had its primative anoxic H_2 _and CO_2 _atmosphere.

**Figure 3:**
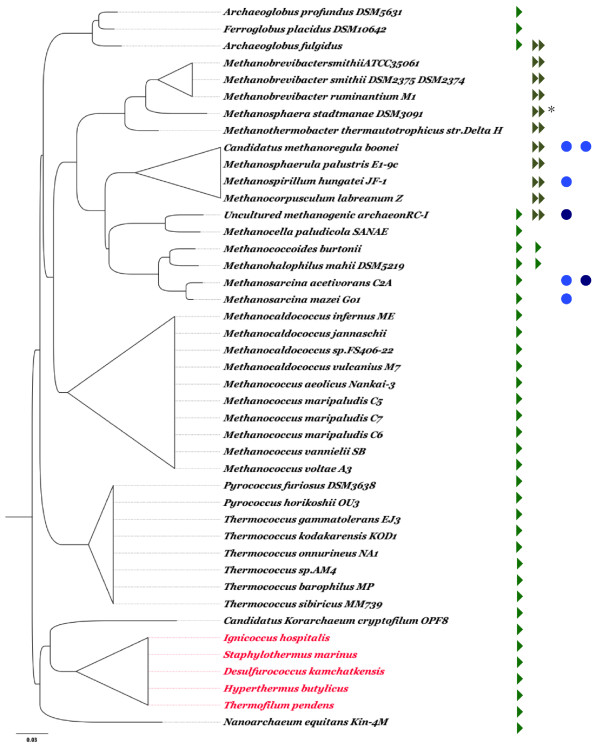
**Repartition of superoxide reductase (SOR) and superoxide dismutase (SOD) genes regarding the 16S rRNA gene distance tree of all archaeal described in SORGOdb**. All of the sequences were retrieved from SILVA [60] when available or GenBank (http://www.ncbi.nlm.nih.gov/). SOR are represented with a single green arrow, Dx-SOR with a double khaki arrow, Fe-Mn SOD by a light blue dot and Cu-Zn SOD by a dark blue dot. SOD-type genes were determined using OxyGene [36]. Scale bar: 3% difference. Crenarchaeota (in red) are developed in Figure 4.

Using the "Browse by phylogeny" option of SORGOdb, we collected the names of all Archaea that possess at least one SOR gene in their complete or partial genomes. Then, we generated a 16S-based phylogenetic tree for these organisms, using ClustalW [46] and sequences recovered from the SILVA comprehensible ribosomal RNA databases [60] (http://www.arb-silva.de/), clustered by Maximum Likelihood and Neighborhood joining algorithms (Neighborhood joining tree is not shown). This tree was annotated with the class of SOR and the presence of SOD on the genome (Maximum Likelihood Tree; Figure 3).

Nanoarchaeota [61] and Korarchaeota [62] are obligately anaerobic sulphur-dependent organisms placed close to the root of the archaeal SSU rRNA tree. Nanoarchaeota is currently known from a single organism *Candidatus Nanoarchaeum equitans*, a hyperthermophilic symbiont that grows on the surface of *Ignicoccus hospitalis *[62,63]. There are currently no representatives of Korarchaeota in pure culture but the genome of *K. cryptophilum*, a very thin filamentous thermophilic heterotroph, has been determined from a sample of Yellowstone National Park Obsidian Pool. Both *C. N. equitans *and *K. cryptophilum *are found together in the 16S tree, in the vicinity of the Crenarchaeota group, and contain genes encoding superoxide reductase with a SOR (centre II) functional domain and do not encode superoxide dismutase genes.

**Figure 4:**
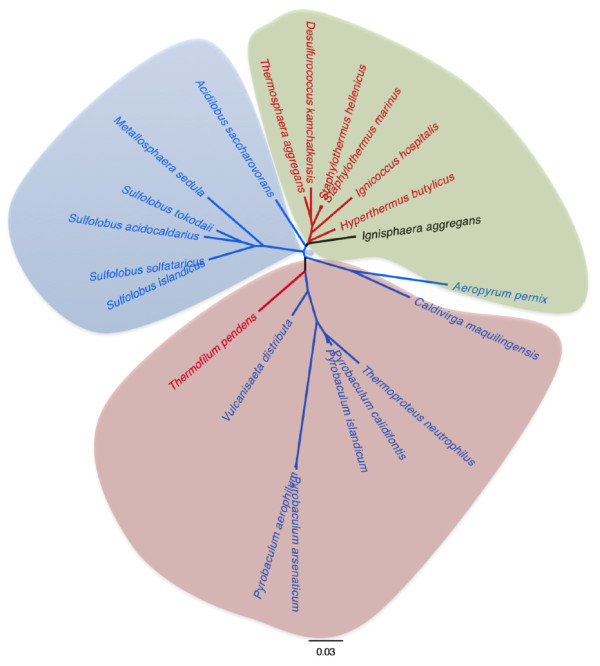
**Repartition of superoxide reductase (SOR) and superoxide dismutase (SOD) genes regarding the 16S rRNA gene distance tree of all Crenarchaeota described in SORGOdb**. All of the sequences were retrieved from SILVA [60] when available or GenBank (http://www.ncbi.nlm.nih.gov/). The Thermoproteales are highlighted in red, the Sulfolobales in blue and the Desulfurococcales in green. Organisms having at least one SOR, or one SOD or none of both (any SOD and any SOR) are respectively represented in red, blue and dark.

According to 16S rRNA gene sequences, the Crenarchaeota group can be subdivided into three orders, the Thermoproteales, the Sulfolobales and the Desulfurococcales [64]. All Sulfolobales and Thermoproteoles genomes studied encode a single SOD, with the single exception of the unique member of the Thermofilaceae familly, *Thermofilum pendens*, an anaerobic commensal that encodes a SOR. By contrast, all Desulfurococcales genomes available encode a SOR but not a SOD, except *Aeropyrum pernix *that has the particularity to be strictly aerobic [65] and that encodes an extremely thermostable Mn/Fe superoxide dismutase [66] and *Ignisphaera aggregans*, a novel deep-branching member of the Desulfurococcaceae lineage of strict anaerobes (as even trace quantities of oxygen inhibited its growth, [67] ) the genome of which carries neither SOR or SOD genes. Other Desulfurococcales studied (Figure 4) have all a gene encoding a centre II mono-domain SOR-type enzyme. Interestingly, two recent genomes have been made available since the last update of SORGOdb (May 2010) and both contain annotation for SOR-like genes: Tagg_0590, described as a Desulfoferrodoxin ferrous iron-binding protein of *Thermosphaera aggregans *DSM 11486 and Shell_0770 for *Staphylothermus hellenicus *DSM 12710, annotated as a twin-arginine secreted superoxide reductase, by homology with *Geobacter metallireducens *GS-15 Gmet_2613 SOR. Using the SORGOdb "search by BlastP", we could confirm that both ORFs are true SOR (ten best e-value from e-59 to e-34) and belong to the SOR-type class. This analysis contradicts the annotation of Shell_0770 in NCBI as TAT-SOR; the absence of a significant TAT targeting signal in Shell_0770 was tested and confirmed by TatFind [68] and TatP [69] predictions. The SORGOdb "search by BlastP" tool therefore allows the accuracy of public SOR annotations to be checked and allows suggestions of their possible SORGOdb classification.

Thermococcus and Pyrococcus are obligate anaerobes that live in environments where there is no oxygen and both produce a SOR-type superoxide reductase that is catalytically active at temperatures below the optimum growth temperature but representing conditions likely corresponding to zones of oxygen exposure [23].

**Figure 5:**
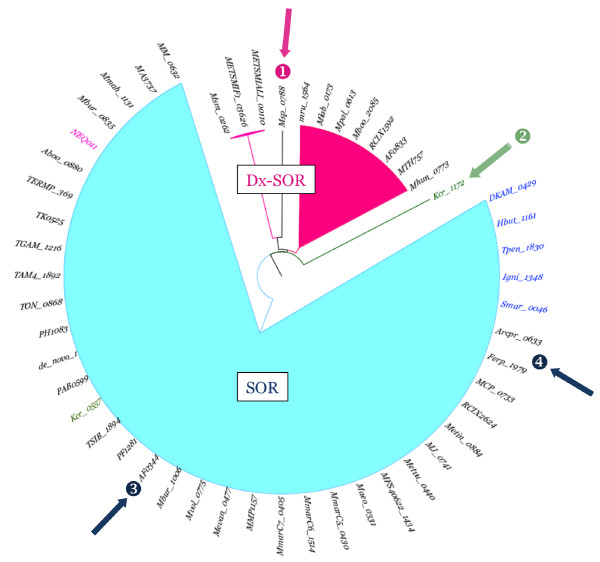
**An evolutionary maximum likelihood tree of archaeal SOR proteins**. The tree shows the repartition of SOR (blue area) and Dx-SOR (pink area) types.

Archaeoglobus is a true archaeal sulphate reducer, reducing SO_4_^2- ^to H_2_S in hot marine sediments. Two complete Archaeoglobus genomes are available, *A. fulgidus *and *A.profundus*, The *A. fulgidus *genome contains one SOR and one Dx-SOR, and the two enzymes have similar kinetics of the superoxide reduction. This raises the question of functional redundancy as Dx-SOR is absent from *A. profundus *and from the related *Ferroglobus placidus*, an iron-oxidising nitrate-reducing species that lives in anoxic (oxygen free) and hot (85°C) environments [70]. The *A. profundus *genome (1.6 Mb) is significantly smaller than those of *A. fulgidus *(2.2 Mb) and *F. placidus *(2.2 Mb). Using the SORGOdb "by organism name search" option, it is easy to compare the genomic locations (GC view map) and the genes contexts (gview synteny map) of the SOR of these three species. This visualization reveals that these genes have different genetic locations and, although the neighbouring genes encode related functions, the genetic organization and order, are not conserved. Again using the "Browse by phylogeny" option of SORGOdb, we get quickly all archaeal SOR amino acid sequences (using check all then get all amino acid sequence) can be selected and used to cluster by Maximum Likelihood using ClustalW to produce a protein distance-tree (Figure 3). This tree shows the position of each four proteins considered (AF0833, AF0344, Arcpr_0633 and Ferp_1979) and indicate that the two *A. fulgidus *SOR (Figure 5, point 3 and 5) are very distant from those of *A. profundum *and *F. placibus*, which by contrast are closely related (Figure 5, point 4). This proximity cannot be linked to the origin of the organisms as *A. fulgidus *and *F. placibus *originate from a shallow marine hydrothermal system at Volcano, Italy [70,71] whereas *A. profundus *was isolated from a deep sea hot vent area (depth: 2000 m) at Guaymas, Mexico [72]. However, based on 16S rRNA gene sequences, indicate that *A. profundus *and *F. placidus *are the most closely related with 96.5% sequence identity.

The protein tree also revealed two interesting phenomena: Msp_0788 that is a non-canonical Dx-SOR (as the Dx active site is incomplete) that is branched as an out-group close to the entire archaeal Dx-SOR group (Figure 5, point 1). This is consistent with the presumed loss-of-function of Dx of Msp_0788 being relatively recent. Also, the Kcr_1172 locus forms a major divergent branch (Figure 5, point 2).). Using the "Browse by locus tag" option, Kcr_1172 is revealed to be a fusion protein with an additional C-terminal module sharing significantly similarities with archaeal proteins annotated as "hypothetical" or "redoxin domain-containing". The best-conserved component is a CXXC motif (i.e. cysteines separated by two amino acids), found in many redox proteins for the formation, the isomerization and the reduction of disulphide bonds and for other redox functions [73]. Kcr_1172 has a new SOR-derived architecture with the presence of two CXXC active sites (in the C-terminal fusion and N-terminal "Dx parts"), separated by the functional SOR centre II. This arrangement is unique and interesting as a combination of two sites CXXC motifs has been shown to be involved in protein disulphide-shuffling in hyperthermophiles [74]. Although the true function of this protein needs to be determined experimentally, we show with this example that SORGOdb can also be used to reveal possible new SOR features.

The distribution of genes encoding SOR and SOD is extremely heterogeneous, both qualitatively and quantitatively, in the group of methanogenic archaea as shown in Figure 3. Thus, for the genus Methanosarcina, *Methanosarcina acetivorans *(5.8 Mb) possesses one SOR and two SOD whereas *Methanosarcina mazei *(4.1 Mb) encodes only one SOR. *M. barkeri*, that shares 80% identity with both *M. acetivorans *and *M. mazei *[75], encodes two SOD [36] but no SOR. The presence of these various combinations of oxygen-dependent SOD and SOR genes confirm that methanogens, that are sensitive to oxygen and are rapidly killed by even very low concentrations of O_2_, protect themselves from ROS; however, the factors that influence the presence and evolution of these genes remain unidentified. No clear relationship can be established between oxygen tolerance and the existence of superoxide reductase functions in the genome of microbes. A difficulty is the different connotations of the term 'anoxia' as used by geologists, zoologists and microbiologists. Geologists call an environment 'aerobic' if the oxygen content exceeds 18%. Zoologists talk about 'hypoxic' conditions when referring to oxygen levels that limit respiration (usually less than ca. 50% O_2_). For microbiologists, the so-called 'Pasteur point' of switch from aerobic respiration to fermentation is generally less than about 1 per cent of the atmospheric levels of oxygen; microbes, though, are affected by very low levels of oxygen, often much less than 0.1 per cent whereas some "anaerobes" living today are able to tolerate oxygen even at higher levels.

## Conclusions

The SORGOdb server is the first web server that centralizes and provides an interface for information concerning superoxide reductase proteins. SORGOdb provides integrated features: (1) Multiple options for data browsing and searching (2) Complete descriptions of SOR and a new domain-based classification (3) Synthetic and downloadable synopsis for each locus tag (4) A SOR-homology analysis tool using BlastP similarity searches with the SORGOdb-positive dataset (5) An integrated access to external hyperlinks to various public data sources (notably NCBI GenBank, and Pubmed). SORGOdb is a unique mining tool that can assist researchers with diverse interests to retrieve, visualize and analyse superoxide reductase genes and proteins.

## Availability and requirements

**Database name**: SORGOdb

**Project home page**: http://sorgo.genouest.org/index.php

**Operating system(s)**: Platform independent, designed for Safari and Firefox browser and not available for Internet Explorer.

**Programming languages**: PHP5 (PHP4 compatible), (X)HTML, CSS2, JavaScript, JQuery, MySQL 5.

## List of abbreviations used

Dfx : Desulfoferrodoxin

Dx : Desulfoferrodoxin

Nlr : Neelaredoxin

ROS : Reactive Oxygen Species

SOD : superoxide dismutase

SOR : superoxide reductase

TAT : Twin-arginine translocation

## Authors' contributions

CLM and FBH jointly carried out the literature survey and designed the study. CLM and FBH retrieved, analyzed, prepared the SOR dataset (sequence, reference, ontology...) and illustrated the relational database. DT and DG performed scripts for automated data retrieval. CLM developed the original web pages and FBH proposed design improvements. DG and CLM worked together on the PHP code. DG conceived the synopsis computation and performed all debugging activities. CLM and FBH wrote the manuscript. FBH managed the project. GS is the Sp@rte team leader and provides CLM financial support. All authors read and approved the final manuscript.

## Supplementary Material

Additional file 1 **Distance trees and alignments for each SORGOdb classes and subclasses**. The Dx-SOR (**Figure A**) and Class II-related SOR (**Figure B**) trees, based on genetic distances, were constructed using ClustalW and UPGMA algorithm. Clade divisions are illustrated by alternatively pink and yellow highlighted area and sequences selected to represent each clade in the alignment are written in red. Multiple sequence alignment were performed using ClustalW and visualized with Jalview [113,114]. Conserved amino acids are highlighted with different shades of blue considering the degree of identity (most conserved amino acids are coloured in dark blue). These alignments correspond to selected Dx-SOR (**Figure C**), selected Class II-related SOR (**Figure D**), all Class III-related SOR (**Figure E**), all Class IV-related SOR (**Figure F**), all TAT-SOR (**Figure G**) and all HTH-Dx-SOR (**Figure H**). Residues that bind the catalytic center are indicated by a blue asterisk. The amino acid sequences corresponding to SOR which have been biochemically characterized are indicated by a blue arrow. The different SOR domains for each class of SOR, are represented just below multiple sequence alignment. Click here for file
