# Supplementary material for: SORGOdb: Superoxide Reductase Gene Ontology curated DataBase
Source: BMC Microbiol. 2011 May 16;11:105. doi: 10.1186/1471-2180-11-105 (PMC3116461; doi:10.1186/1471-2180-11-105)
Supplement: Additional file 1 — Distance trees and alignments for each SORGOdb classes and subclasses. The Dx-SOR (Figure A) and Class II-related SOR (Figure B) trees, based on genetic distances, were constructed using ClustalW and UPGMA algorithm. Clade divisions are illustrated by alternatively pink and yellow highlighted area and sequences selected to represent each clade in the alignment are written in red. Multiple sequence alignment were performed using ClustalW and visualized with Jalview [113,114]. Conserved amino acids are highlighted with different shades of blue considering the degree of identity (most conserved amino acids are coloured in dark blue). These alignments correspond to selected Dx-SOR (Figure C), selected Class II-related SOR (Figure D), all Class III-related SOR (Figure E), all Class IV-related SOR (Figure F), all TAT-SOR (Figure G) and all HTH-Dx-SOR (Figure H). Residues that bind the catalytic center are indicated by a blue asterisk. The amino acid sequences corresponding to SOR which have been biochemically characterized are indicated by a blue arrow. The different SOR domains for each class of SOR, are represented just below multiple sequence alignment. [file 1471-2180-11-105-S1.PDF]

Fig. A
Dx-SOR distance tree

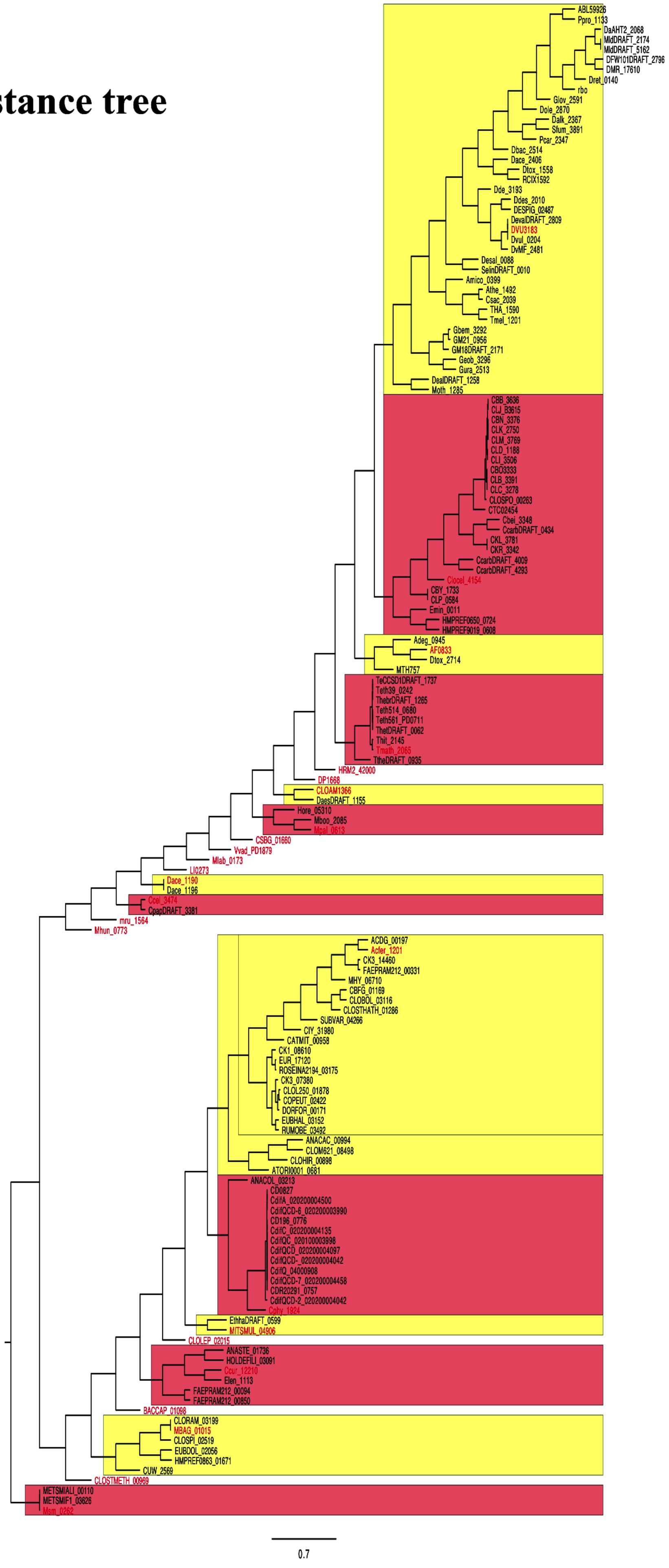

Fig. B
Class II-related distance tree

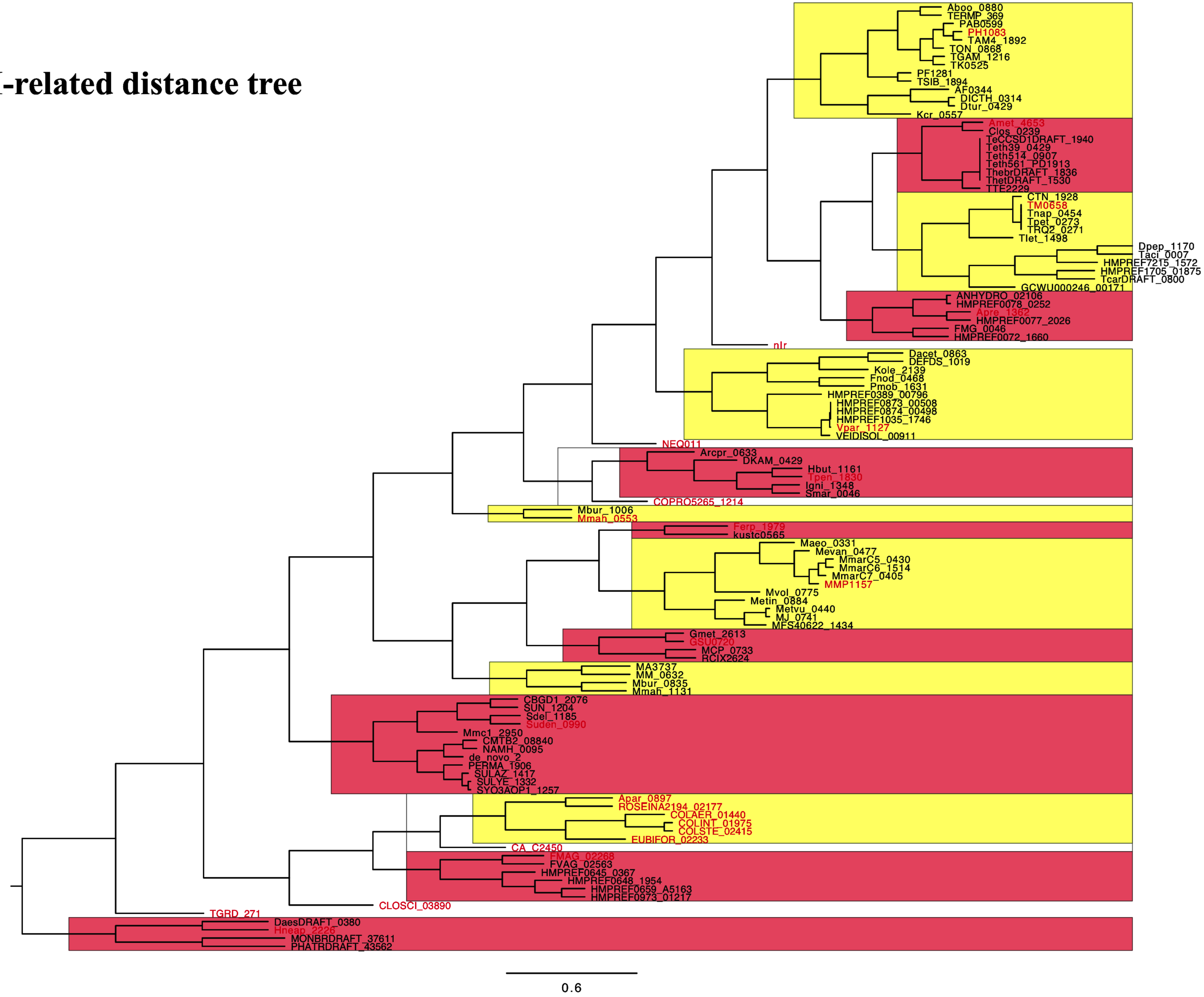

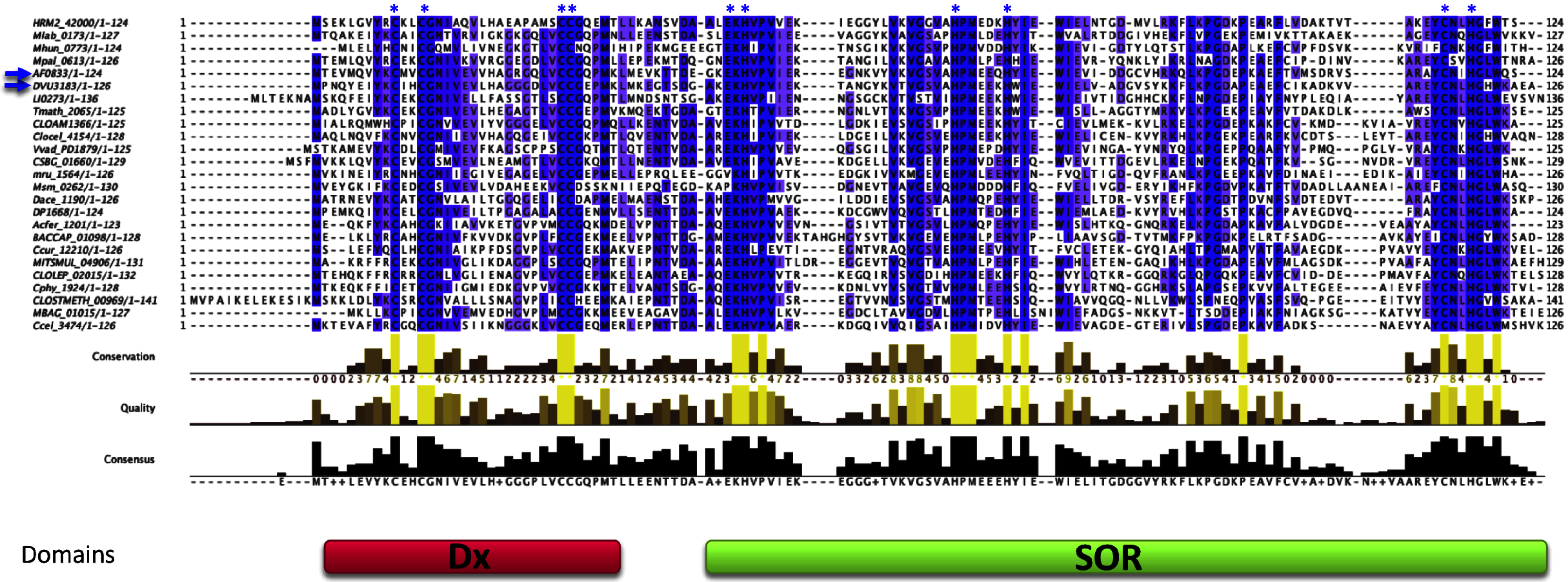

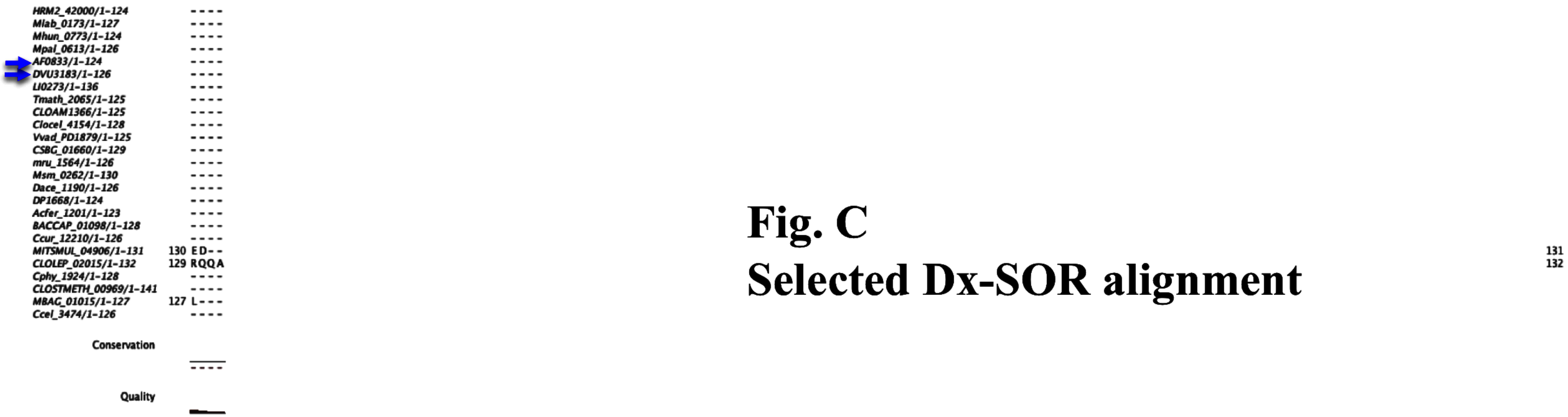

Fig. C
Selected Dx-SOR alignment

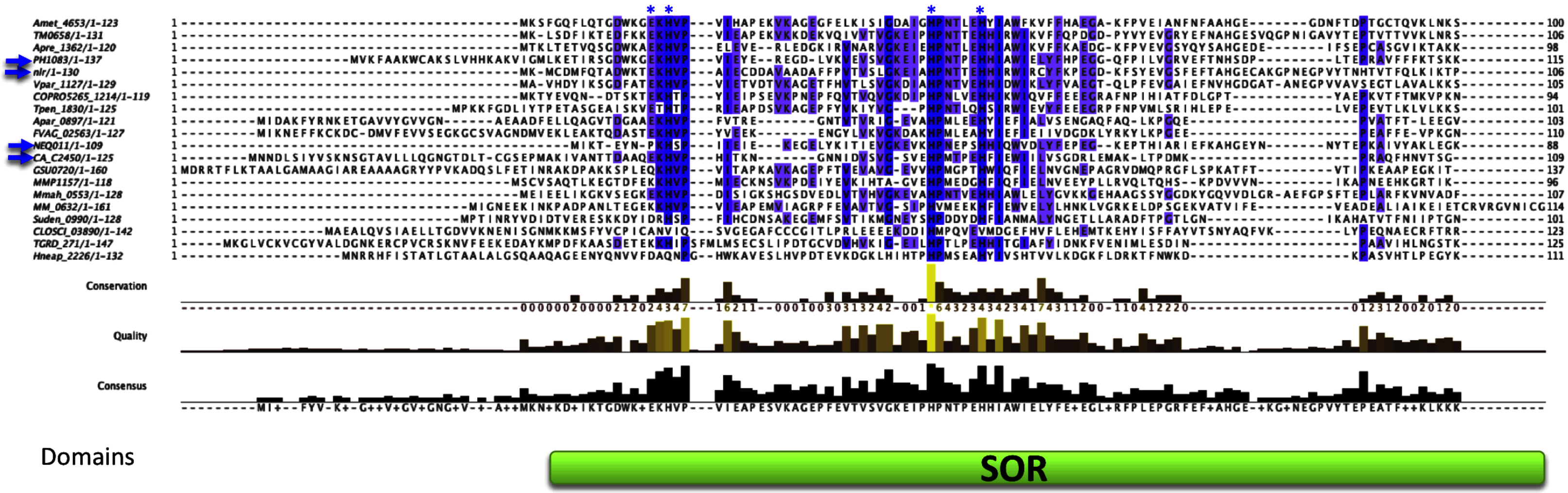

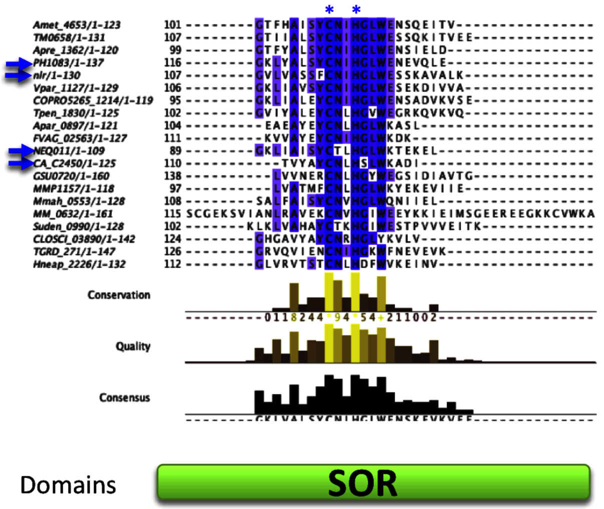

Fig. D

Selected Class II-related SOR alignment

Fig. E
Class III-related SOR alignment

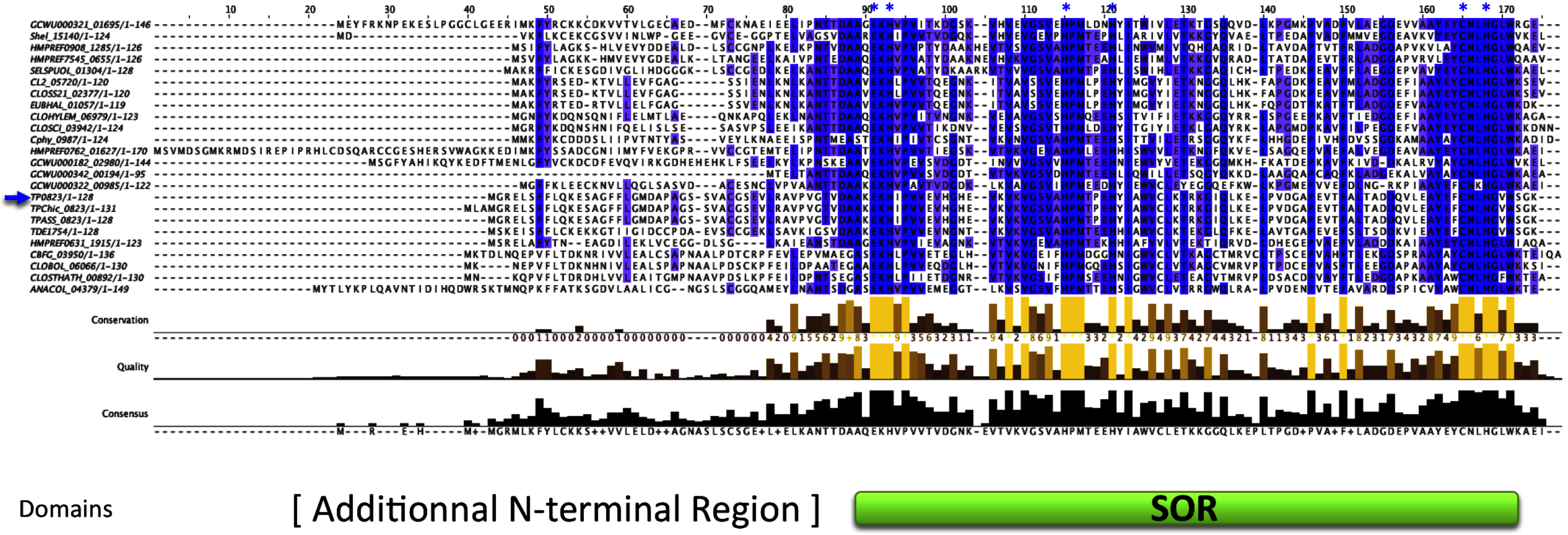

Fig. F
Class IV-related SOR alignment

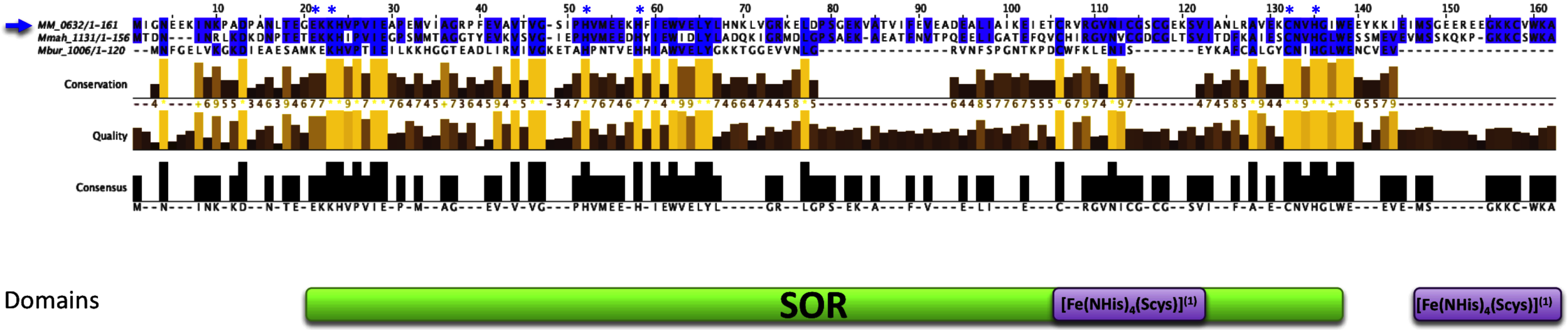

(1) Amino acids forming the $[Fe(NHis)_4(Scys)]$

Fig. G
TAT-SOR alignment

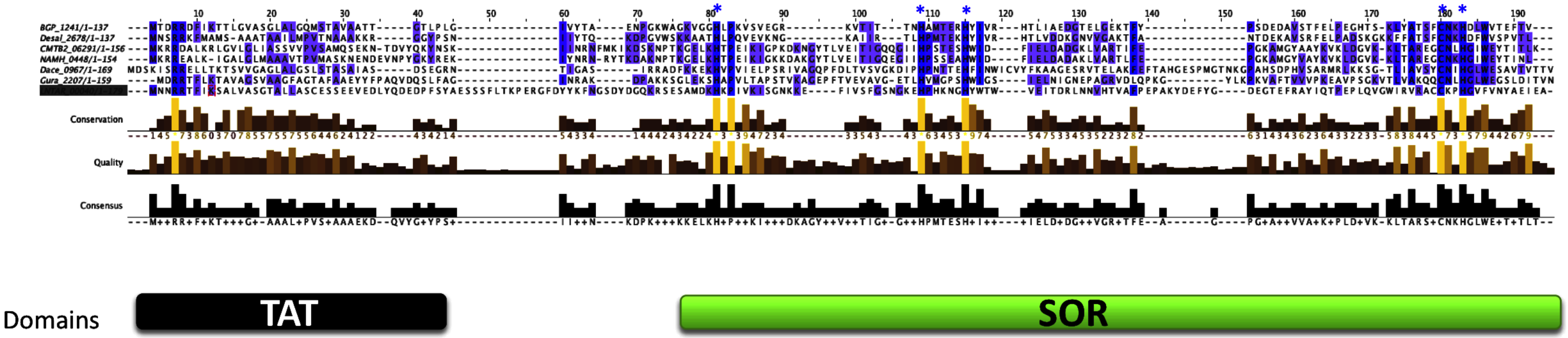

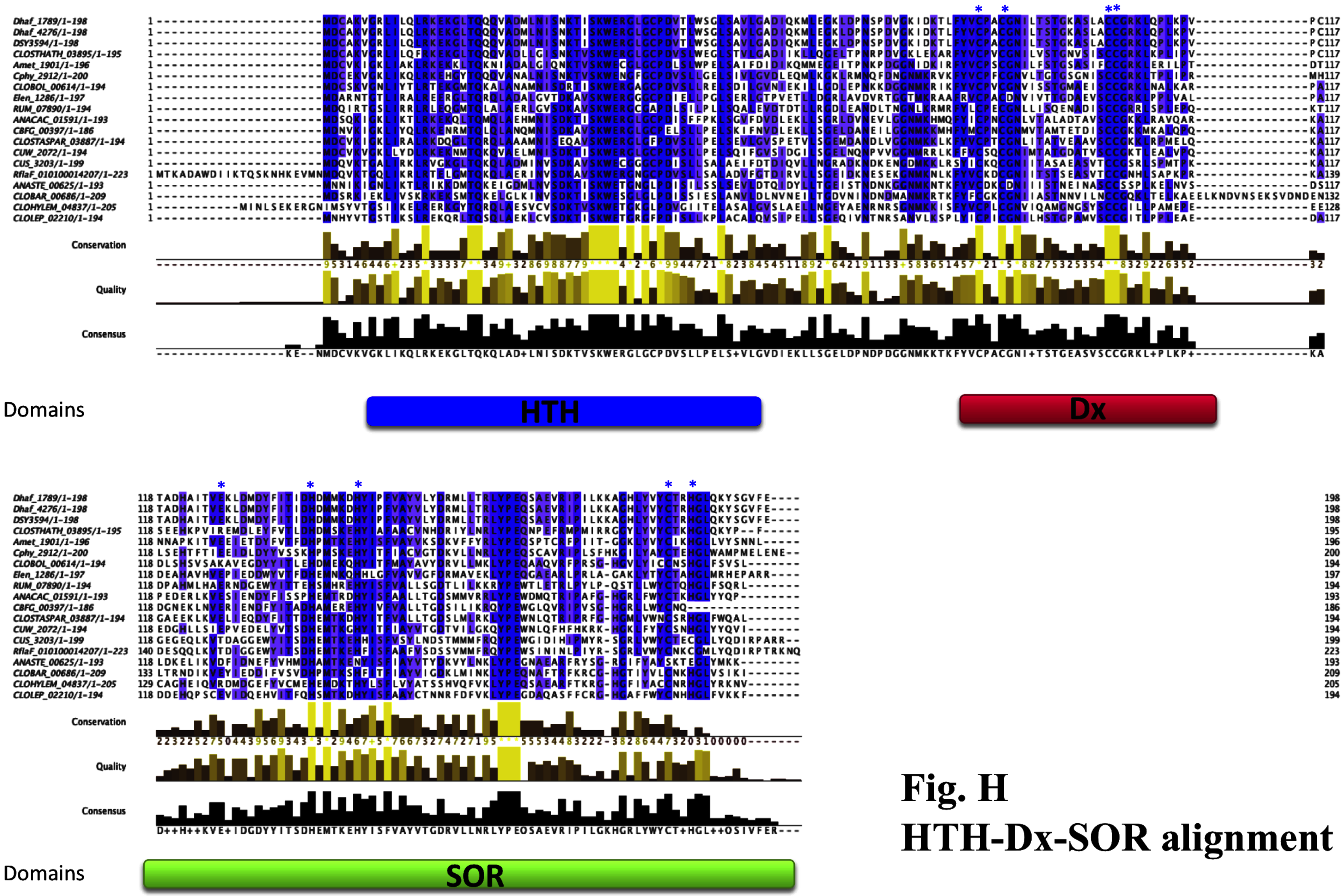

Fig. H
HTH-Dx-SOR alignment
